# Association between self-reported vegetarian diet and the irritable bowel syndrome in the French NutriNet cohort

**DOI:** 10.1371/journal.pone.0183039

**Published:** 2017-08-25

**Authors:** Camille Buscail, Jean-Marc Sabate, Michel Bouchoucha, Marion J. Torres, Benjamin Allès, Serge Hercberg, Robert Benamouzig, Chantal Julia

**Affiliations:** 1 Equipe de Recherche en Epidémiologie Nutritionnelle, Université Paris 13, Centre de Recherche en Epidémiologie et Biostatistiques (CRESS), Inserm 1153, Inra U1125, Cnam, COMUE Sorbonne Paris Cité, Bobigny, France; 2 Département de santé publique, Hôpital Avicenne, Bobigny, France; 3 Service d’ Hépato-Gastro-Entérologie, Hôpital Avicenne, Bobigny, France; University Hospital Llandough, UNITED KINGDOM

## Abstract

**Background:**

There is growing interest in using diet counselling in the management of patients with irritable bowel syndrome (IBS). Among new emerging diets, vegetarian diets (VD) seem to be experiencing an important popularity, partly because of their alleged health benefits. A recent study performed among a rural Indian population showed that predominant VD could be associated with IBS.

**Objective:**

This cross-sectional study aimed at assessing the association between the VD and IBS, among a large French cohort, the NutriNet-santé study.

**Methods:**

Subjects participating in the NutriNet-Santé cohort study completed a questionnaire based on Rome III criteria (N = 41,682). Anthropometrics, socio-demographical and lifestyle data, including VD, were collected prior to the completion of Rome III questionnaire via self-administered questionnaires. Association between VD and IBS and its subtypes was investigated through multivariate logistic regression.

**Results:**

The included subjects were mainly women (78.0%) and the mean age was 49.8±14.3 years. Among these individuals, 2,264 (5.4%) presented an IBS, and 805 (1.9%) reported a VD. Overall, VD was not associated with IBS or subtypes. A stable VD (i.e. self-declared at least three times) was associated with IBS (aOR 2.60 95%CI [1.37–4.91]), IBS mixed (aOR 2.97 95%CI [1.20–7.36]) and IBS diarrhoea (aOR 2.77 95%CI [1.01–7.59]).

**Conclusions:**

This study suggests that a long term VD could be associated with IBS. Nevertheless, further studies are needed to confirm these results, and investigate the multiple aspects of the vegetarian diet, possibly related to the IBS.

## Introduction

Vegetarian diet (VD), that includes the partial or total removal of meat, poultry, fish from the diet, (vegans also exclude dairy products and eggs), is increasingly widespread among the general population [1–4]. The reasons for adopting this dietary profile are attributable to ethical, environmental, and social concerns [1,2,5–10]. Health aspects of such a diet are also more and more emphasized. Indeed, health benefits of the VD, especially on ischemic heart disease and cancer have been widely reported by cross-sectional and prospective cohort studies during the last 50 years [11–14]. Generally speaking, vegetarians tend to be more health conscious, with a lower body mass index (BMI), and in better health when compared with omnivores, giving this type of diet a clear appeal in the population of subjects suffering from chronic diseases [15]. Furthermore, several health crises surrounding meat erupted in recent years (including animal bone meal or mad cow disease), and the world health organisation (WHO) has classified in 2015 red meat and processed meat as *Group 2A*, that is "probably carcinogenic" to humans [16]. Finally, VD patterns (in comparison to meat-based diets) are more sustainable because they use substantially less natural resources and are less taxing on the environment [17–19]. Adopting a VD may therefore seem a beneficial diet in many ways in the future.

Irritable bowel syndrome (IBS) is one of the most common functional gastrointestinal disorder (FGID), defined by abdominal pain and abnormal transit pattern, with the absence of detectable organic illness [20–22]. Prevalence of IBS in the industrialized world is estimated to be approximately 12%, which makes IBS one of the most common gastrointestinal disorder [23]. Among several factors supposed to be involved in the pathogenesis of IBS, diet appears to play a key role [24–29]. Two thirds of IBS patients (64%) report meal-related symptoms to at least one food item [24], and they therefore often express an intense interest in food choice and attempt to identify and remove foods that induce symptoms [30–32]. For example, a cross-sectional study showed that 62% of IBS patients limited or excluded some food items from the diet [24]. Given the lack of scientific evidence supporting specific dietary advice for patients with IBS, they tend to adopt new diets, guided by various way of life (empirical, philosophical, etc), and spread via the media [29,33]. These changes include exclusion diets like VD. Adopting a VD pattern could induce some effects on the digestion process [34–36], and even on digestive diseases: Crowe and colleagues have shown that consuming a VD and a high intake of dietary fibre were both associated with a lower risk of admission to hospital or death from diverticular disease [37]. Moreover, the beneficial effects of a VD on inflammatory bowel diseases (and in particular the prevention of relapses) are increasingly considered [38,39].

A recent cross-sectional study performed among the rural Indian population found that participants with a predominant VD were more at risk for having IBS than those with a non-VD [40]. However, to the best of our knowledge, the association between a VD and IBS has not been studied to date among occidental populations.

This study aimed to assess the association between the vegetarian diet and IBS among a large French sample included in the NutriNet-Santé study.

## Methods

### Population

The *NutriNet-Santé* Study is a web-based prospective observational cohort. It aims to investigate the relationships between health and nutrition. The inclusion of subjects aged over 18 years started in France in May 2009 and still ongoing with 158,361 subjects enrolled at the time of the study. At baseline, participants completed self-administered questionnaires about socio-economic, lifestyle, health status, diet, physical activity, and anthropometrics data. During follow-up, additional questionnaires are regularly performed in accordance with the declaration of Helsinki and were approved by the institute Review Board of the French Institute for Health and Medical Research (00000388FWA00005831) and the Commission Nationale de l’Informatique et des Libertés (CNIL 908450 and 909216). All participants provided an electronic informed consent.

### Data collection

#### Irritable bowel syndrome

A questionnaire assessing presence of FGIDs was sent to the whole cohort on 21 June 2013, and the questionnaire was available for completion up to the 6 November 2013, including data on medical digestive history and symptoms using the Rome III criteria. IBS and subtypes of the disease (IBS-Constipation, IBS-C, IBS-Diarrhea, IBS-D, IBS-Mixed, IBS-M and IBS-undefined, IBS-U), were defined according to the Rome III criteria and had to be present at least for the last 6 months [41,42]. Subjects reporting any organic diseases (stomach, esophagus or colorectal cancers, familial adenomatous polyposis coli, Crohn’s disease, coeliac disease, ulcerative colitis) or alarm symptoms (melena, hematemesis, rectal bleeding or significant unintentional weight loss in the past 3 months), were excluded from the present study.

#### Dietary data

At baseline and every 6 months, participants were requested to complete web based self-administered 24h dietary records. All participants who completed at least three 24h-records before the completion of the questionnaire pertaining to FGIDs were eligible. Each food and beverage consumed was collected according to the three main meal (breakfast, lunch and dinner) and possibilities of snack. Participants had to estimate the portion size for each elements consumed using validated photographs [43]. Dietary intake was estimated using the NutriNet-Santé food composition table, including more than 2,500 different foods and estimating total energy intake. Average energy intake from all dietary questionnaires was took into account in multivariate analysis as a covariate.

#### Vegetarian diet

Information on VD was collected at baseline and annually through follow-up questionnaires, using the following interrogation: “Currently, do you follow a particular diet? (Medical, pregnancy, vegetarian, vegan, personal or religious conviction)”. In this study we considered self-reported vegetarian diet was considered as a good proxy for vegetarianism. Thus, anyone reporting at least once following a VD was considered vegetarian. We also took into account “stable” vegetarians, i.e. participants who declared at least 3 times they followed a VD (whether at baseline or throughout the follow-up questionnaires) in Nutrinet for analyses. Since they represent a very particular population, we excluded participants who declared they followed a vegan diet (n = 226), but they were considered for sensitivity analyses.

#### Covariates

At baseline, information on age, gender, BMI (normal/overweight or obese), smoking status (current smoker/former smoker/nonsmoker), marital status (single/living in couple), income level (<1200 € per consumer unit (c.u.)/1200-2300 € per c.u./>2300 € per c.u.) and educational level (no diploma or primary studies/secondary studies or higher educational level) were collected by self-administered questionnaire. Physical activity (PA) level was assessed using International Physical Activity Questionnaire (IPAQ) at baseline, and was divided into three categories according to the mean MET per week [44] as follows: PA was low when mean MET per week was less than 1500, moderate when PA was upper than 1500 and less than 3000, and high above 3000 MET [44].

### Statistical analyses

A description of socio-demographical, lifestyle, anthropometrical and medical information was performed according to the gender with t-tests and chi-square tests, according to the type of variable. Comparison of food group consumption and nutrient intake between vegetarians and omnivores was also realized, controlled for gender, age and total energy intake (ANCOVA tests). Interactions according to the IBS status were tested. Multivariate logistic regression models were performed to estimate the association between VD and IBS and subtypes, adjusting for the known or suspected risk factors listed above. Among these factors, those clearly identified in the literature were forced into the models (ie. age, educational level, smoking status, BMI and physical activity), additional factors associated with IBS with p<0.05 in bivariate analyses were included. Results of logistic regression models are presented using adjusted Odds Ratio (aOR) and 95% Confidence Interval (95% CI). To handle missing data of two covariates, multivariate logistic regression models were performed using multiple imputation [45,46]. Imputed values for physical activity (missing data = 5,290, 12.6%) and income level (missing data = 4,446, 10.7%) were estimated conditionally on the following variables: age, gender, marital status and educational level. A second model was performed, with the stable vegetarians as outcome. Finally, we performed sensitivity analyses through multivariate logistic regression models by including vegans in the definition of the outcome. Statistical analyses were conducted using SAS statistical package release 9.4 (SAS institute, Inc., Cary, NC, USA).

## Results

**Fig 1:**
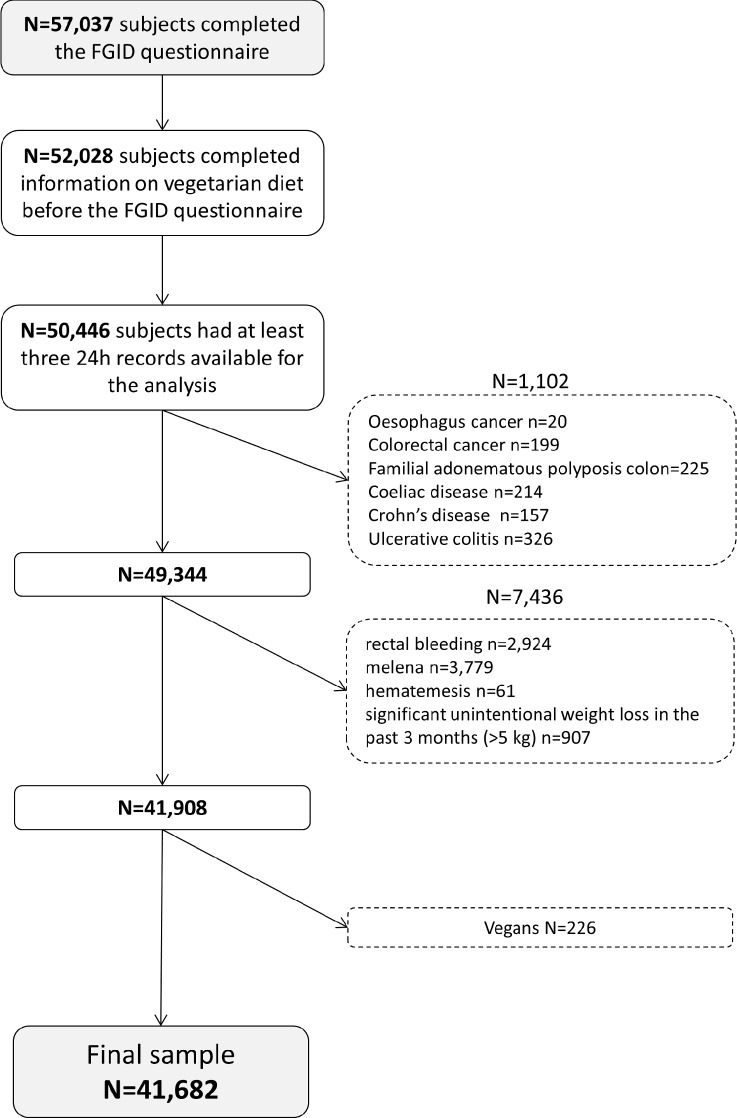
Flowchart of the study.

In the NutriNet-Santé Study, 57,037 individuals filled the FGIDs questionnaire. Among them, 52,028 completed information on VD before answering the FGIDs questionnaire. Among them, 50,446 subjects had at least three 24H records available for analysis. The 8,538 participants that reported any organic diseases (stomach, esophagus or colorectal cancers, familial adenomatous polyposis coli, Crohn’s disease, coeliac disease, ulcerative colitis) or alarm symptoms (melena, hematemesis, rectal bleeding or significant unintentional weight loss in the past 3 months) were excluded. Finally, we excluded participants who declared themselves vegans (n = 226) from the analyses (see **Fig 1**).

Comparison according to the main characteristics between included subjects (n = 41,682) and those removed (n = 8,784) is shown in **S1 Table**. Excluded subjects were younger, more often men, current smoker, single with a lower income. The final sample included 41,682 subjects. Subjects included were mainly women (78.0%) and the mean age was 49.8 +/-14.3 years. Overall 2,264 (5.4%) subjects reported an IBS, with a higher prevalence in women compared to men (5.6% vs 4.8%, p = 0.03) (**Table 1**). Prevalence of IBS subtypes were distributed as follows: 2.0% (n = 847) for IBS-M, 1.7% (n = 727) for IBS-D, 1.2% (n = 478) for IBS-C and 0.5% (n = 212) for IBS-U, with a higher prevalence in women for IBS-c and IBS-u (**Table 1**). Overall 1.9% (n = 805) subjects declared they followed a VD, mostly women (2.1% vs 1.4%, p<0.001). The proportions of vegetarians in IBS group and control group were similar (respectively 1.9% vs 2.0% with p = 0.84) (**Table 2**).

**Table 1:** Description of IBS and subtypes prevalence according to gender (N = 41,682).

	Total	Men n = 9,184		Women n = 32,498		p*
		(22.0%)		(78.0%)		
	N (%)	n	%	n	%	
**IBS**						<0.01
No	39,418 (94.6%)	8,742	95.2	30,676	94.4	
Yes	2,264 (5.4%)	442	4.8	1,822	5.6	
**IBS Mixed**						
No	40,835 (98.0%)	8,990	97.9	31,845	98.0	0.54
Yes	847 (2.0%)	194	2.1	653	2	
**IBS Diarrhoea**						
No	40,955 (98.3%)	9,010	98.1	31,945	98.3	0.21
Yes	727 (1.7%)	174	1.9	553	1.7	
**IBS Constipation**						
No	41,204 (98.8%)	9,133	99.4	32,071	98.7	<0.0001
Yes	478 (1.2%)	51	0.6	427	1.3	
**IBS Undefined**						
No	41,470 (99.5%)	9,161	99.7	32,309	99.4	<0.0001
Yes	212 (0.5%)	23	0.3	189	0.6	

* Chi-square tests were performed

**Table 2:** Comparison of sample characteristics between healthy controls and IBS patients (N = 41,682).

	Controls n = 39,418 (94.6%)		IBS n = 2,264 (5.4%)		p*
	N	%	N	%	
**Gender**					<0.01
Men	8,742	22.2	442	19.5	
Women	30,676	77.8	1,822	80.5	
**Age (mean +/-SD)**	49.5	14.3	56.0	11.9	<0.0001
**Marital status**					
Single	10,531	26.7	609	26.9	0.85
Couple	28,887	73.3	1,655	73.1	
**Education level**					
No diploma and primary studies	1,134	2.9	78	3.4	<0.01
Secondary studies	13,011	33.0	806	35.6	
High educational level	25,273	64.1	1,380	61.0	
**Income level**					
<1200 €	5,460	15.5	258	12.7	<0.001
1200–2300 €	15,404	43.4	855	42.1	
> 2300 €	14,339	40.7	920	45.2	
**Smoking status**					
Non smoker	20,456	51.9	1,095	48.4	<0.0001
Former smoker	13,783	35.0	924	40.8	
Smoker	5,179	13.1	245	10.8	
**Physical activity**					
Low	7,587	22.1	432	21.4	0.57
Moderate	14,766	43.0	858	42.5	
High	12,021	35.0	728	36.1	
**BMI (kg/cm**^**2**^**)**					
< 25	27,610	70.0	1,532	67.7	0.05
25–30	8,535	21.7	525	23.2	
≥ 30	3,271	8.3	207	9.1	
**Vegetarian diet**	38,658	98.1	2,219	98.0	
	760	1.9	45	2.0	0.84

* Chi-square tests were performed in order to compare the proportions of each covariate between IBS and controls

Abbreviations: BMI Body Mass Index; IBS Irritable Bowel Syndrome; SD Standard deviation

Missing data: Income level n = 4,446 (10.7%); Physical activity n = 5,290 (12.6%)

**Table 3**shows the comparison of the mean food consumption between vegetarians and omnivore subjects, adjusted for age, gender and total energy intake. As expected, compared to omnivorous, vegetarians had significantly lower consumption of meat, poultry, fish and shellfish, processed meat and fish.

**Table 3:** Comparison of daily intake of food groups (in grams/day) between vegetarians and omnivores (n = 41,682).

	Omnivores		Vegetarians		
	n = 40,877		n = 805		
	Mean	SE	Mean	SE	p*
Meat, poultry	**97.0**	0.6	37.5	3.7	<0.0001
Porc ham, poultry cuts, processed meat	**43.4**	0.3	17.6	2.1	<0.0001
Fish, shellfish, processed fish and shellfish	**62.9**	0.5	52.5	3.4	<0.01
Eggs	18.8	0.2	**23.3**	1.4	<0.01
Milk, yogurt	155.8	1.6	137.3	10.2	0.07
Cheese, cottage cheese, Petits Suisses	72.2	0.7	**85.3**	4.3	<0.01
Starchy food	239.3	1.0	230.3	6.8	0.18
Wholegrain products	50.1	0.6	**86.6**	4.2	<0.0001
Breakfast cereals	24.2	0.4	**45.4**	2.4	<0.0001
Dry fruits, oleaginous fruits	9.0	0.2	**16.1**	1.2	<0.0001
Fruits	194.3	1.4	**230.3**	9.4	<0.001
Vegetables	214.2	1.1	**273.1**	7.5	<0.0001
Pulses	20.9	0.3	**37.3**	2.2	<0.0001
100% legumes and fruits juice	69.6	0.9	67.7	5.9	0.75
Condiments, spices	9.9	0.1	**11.7**	0.6	<0.01
Oil	9.5	0.1	**11.2**	0.5	<0.01
Non sugared beverages	1,094.8	5.8	**1,257.5**	38.2	<0.0001
Soft sugary drinks	56.6	1.0	**74.4**	6.6	<0.01
Alcoholic beverages	**127.2**	1.5	103.0	9.5	0.01
Fat products	39.7	0.2	38.0	1.4	0.22
Fat and sugared products	177.5	1.0	169.7	6.5	0.24
Salty and sweet snack products	21.6	0.2	20.6	1.5	0.53

*ANCOVA tests controlled for gender, age and total energy intake

Abbreviations: SE: Standard Error

Vegetarians also had lower consumption of soft sugary drinks and alcoholic beverage, while they had significantly higher consumption of eggs, fruits and vegetables, wholegrain products, pulses, cereals, dry fruits, legumes, oil and non-sugared beverages. **Table 4**summarizes the mean daily intake in terms of macronutrients in vegetarians and omnivorous subjects controlled for gender, age and total energy intake. The vegetarians reported lower total energy intake, with lower percent energy from fat and proteins, and higher percent energy from carbohydrates. Vegetarians had lower daily intakes of saturated fatty acids (SFA), cholesterol and animal proteins and they tended to reach the recommended level in fibres (>25g/day) more often than omnivorous. Conversely, they had higher intakes poly unsaturated fatty acids (PUFA), Omega 3, Omega 6 and vegetal proteins. Dietary and nutritional intakes were compared between participants who declared themselves vegetarians at least three times in Nutrinet and others (**S2 Table and S3 Table**). Consistent vegetarians had lower intakes of meat, poultry, and processed meat, and higher intakes of fruits and vegetables. Compared to “simple” vegetarians, consistent vegetarians had also higher intakes of fibres and simple carbohydrates and lower intakes of animal proteins and cholesterol.

**Table 4:** Comparison of daily intake of macronutrients between omnivorous and vegetarians (N = 41,682).

	Omnivorous n = 10,877		Vegetarians n = 805		
	Mean	SE	Mean	SE	p*
Energy (Kcal)	**2014.8**	2.3	1955.2	13.6	<0.0001
%energy from fat	37.9	0.0	37.5	0.2	0.03
MUFA (g)	29.3	0.0	**29.6**	0.2	0.24
Omega 3 (g)	1.4	0.0	**1.5**	0.0	<0.0001
Omega 6 (g)	9.1	0.0	**10.6**	0.1	<0.0001
PUFA (g)	11.1	0.0	**12.8**	0.1	<0.0001
SFA (g)	**32.1**	0.0	30.0	0.2	<0.0001
Cholesterol (mg)	**312.8**	0.6	260.0	3.2	<0.0001
%energy from protein	**16.9**	0.0	14.6	0.1	<0.0001
Animal proteins (g)	**54.8**	0.1	37.0	0.5	<0.0001
Vegetal proteins (g)	24.8	0.0	**31.8**	0.2	<0.0001
%energy from carbohydrates	41.5	0.0	**44.8**	0.2	<0.0001
Complex carbohydrates (g)	105.0	0.1	**112.4**	0.8	<0.0001
Simple carbohydrates (g)	89.0	0.1	**95.8**	0.8	<0.0001
Fibers (g)	19.3	0.0	**24.7**	0.2	<0.0001

* ANCOVA tests controlled for gender, age and total energy intake except for energy, lipids, proteins and carbohydrates

Abbreviations: Kcal: kilocalories; MUFA: MonoUnsaturated Fatty Acids; PUFA: PolyUnsaturated Fatty Acids; SE: Standard Error; SFA: Saturated Fatty Acids

Consumption of calcium, iron, potassium, magnesium, beta carotene, Vitamins A, B1, B6, B9, C and E were significantly higher in vegetarians compared to omnivores (**Table 5**). Other micronutrients were significantly higher in omnivores, especially Sodium, and Vitamin D and Vitamin B12.

**Table 5:** Comparison of daily intake of micronutrients between omnivorous and vegetarians (N = 41,682).

	Omnivores n = 40,877		Vegetarians n = 805		
	Mean	SE	Mean	SE	p*
Calcium (mg)	911.4	1.37	**965.5**	7.77	< .0001
Iron (mg)	13.3	0.02	**15.2**	0.12	< .0001
Potassium (mg)	2972.4	3.48	**3144.1**	19.8	< .0001
Magnesium (mg)	335.7	0.54	**402.4**	3.07	< .0001
Sodium (mg)	**2769.2**	3.51	2510.6	19.96	< .0001
Zinc (mg)	**10.8**	0.01	9.9	0.08	< .0001
Phosphorus (mg)	1262.6	1.42	1276.8	8.11	0.08
Vit A (mg)	1059.4	3.8	**1140.3**	21.7	<0.001
Beta Carotene (μg)	3361.0	13.1	**4428.2**	74.3	< .0001
Vit B1 (mg)	1.1	0.0	**1.2**	0.0	0.02
Vit B2 (mg)	1.7	0.0	1.7	0.0	0.64
Vit B5 (mg)	5.3	0.0	5.2	0.0	0.11
Vit B6 (mg)	1.7	0.0	**1.8**	0.0	<0.001
Vit B9 (μg)	320.1	0.5	**380.2**	3.0	< .0001
VitB12 (μg)	**5.4**	0.0	3.9	0.1	< .0001
Vit B3 (mg)	**18.9**	0.0	16.8	0.2	< .0001
Vit C (mg)	112.5	0.4	**123.3**	2.1	< .0001
Vit D (μg)	**2.7**	0.0	2.6	0.1	0.02
Vit E (mg)	11.2	0.0	**13.3**	0.1	< .0001

* ANCOVA tests controlled for gender, age and total energy intake

Abbreviations: SE: Standard Error; Vit: Vitamin

No significant association was observed between vegetarians and IBS (**Table 6**). When studying vegetarians who declared at least three times they followed a VD, significant associations were shown with IBS (aOR 2.60 95%CI: 1.37–4.91), with IBS mixed (aOR 2.97 95%CI: 1.20–7.36), and with IBS-diarrhoea (aOR 2.77 95%CI: 1.01–7.59). Sensitivity analyses, including vegans showed similar results, plus an association between VD and IBS diarrhoea (aOR 1.55 95%CI: 1.02–2.34) (**Table 7**). Supplementary analyses, with models performed with one outcome splitting our population in three categories (according to the number of self-declarations of VD in Nutrinet, 0, 1 or 2 and at least 3) were performed (**S4 Table and S5 Table**).

**Table 6:** Multivariate analyses (logistic regression models) (N = 41 682).

		Vegetarians (n = 805)	Stable vegetarians (n = 106)
		aOR (95%CI)	aOR (95%CI)
IBS	Omnivorous	Ref.	Ref.
	Vegetarians	1.19 [0.87–1.62]	**2.60 [1.37–4.91]**
IBS mixed	Omnivorous	Ref.	Ref.
	Vegetarians	1.27 [0.79–2.05]	**2.97 [1.20–7.36]**
IBS diarrhoea	Omnivorous	Ref.	Ref.
	Vegetarians	1.32 [0.80–2.18]	**2.77 [1.01–7.59]**
IBS constipation	Omnivorous	Ref.	Ref.
	Vegetarians	1.01 [0.50–2.06]	2.25 [0.55–9.26]
IBS undefined	Omnivorous	Ref.	Ref.
	Vegetarians	0.75 [0.24–2.37]	NA

Models are adjusted for: Age, educational level, total energy intake, income level, smoking status, BMI, physical activity and gender

Abbreviations: IBS Irritable Bowel Syndrome; NA Not Applicable; OR Odds Ratio; 95%CI Confidence Interval

**Table 7:** Multivariate analyses including vegans (logistic regression models) (N = 41 908).

		Vegetarians or vegans (n = 1,031)	Stable vegetarians or vegans (n = 134)
		aOR (95%CI)	aOR (95%CI)
IBS	Omnivorous	Ref.	Ref.
	Vegetarians	1.24 [0.95–1.62]	**2.66 [1.51–4.68]**
IBS mixed	Omnivorous	Ref.	Ref.
	Vegetarians	1.21 [0.78–1.86]	**2.85 [1.24–6.54]**
IBS diarrhoea	Omnivorous	Ref.	Ref.
	Vegetarians	**1.55 [1.02–2.34]**	**3.38 [1.47–7.74]**
IBS constipation	Omnivorous	Ref.	Ref.
	Vegetarians	1.09 [0.60–2.00]	1.77 [0.43–7.24]
IBS undefined	Omnivorous	Ref.	Ref.
	Vegetarians	0.59 [0.19–1.86]	NA

Models are adjusted for: Age, educational level, income level, total energy intake, smoking status, BMI, physical activity and gender

Abbreviations: IBS Irritable Bowel Syndrome; NA Not Applicable; OR Odds Ratio; 95%CI Confidence Interval

## Discussion

A VD was associated with IBS, IBS-M and IBS-D in consistent vegetarians, i.e. when participants declared at least three times they were vegetarians in the Nutrinet study. To the best of our knowledge, this work is the first to specifically assess the relationship between vegetarianism and IBS in such a large population-based study. Numerous approaches to dietary management of IBS have been investigated [30–32,47–52], including an increase of dietary fibre intakes [53–57], identification and management of lactose intolerance [58], and more recently exclusion of food containing Fermentable Oligo-, Di-, Monosaccharides And Polyols FODMAPs [59]. Given this knowledge, some particular features related to a VD could worsen or improve IBS symptoms. In accordance with previous studies performed on vegetarians, our work highlighted that VD provides relatively large amounts of cereals, pulses, nuts, fruits, vegetables and wholegrain products, strengthening the validity of the vegetarians declaration in our sample [60,61]. Vegetarians have therefore i) higher fibres intake, ii) a greater proportion of energy from carbohydrates and iii) lower intake of lactose. The role of dietary fibres on IBS is complex. Despite years of advising patients to increase their global dietary fibre intakes, recent reviews suggest that the benefit of fibres in IBS appears to be limited to soluble fibres [57,62,63].

An increased proportion of certain types of carbohydrates in diet can also worsen IBS symptoms. In particular sugars (mono and disaccharides) and polyols which are slowly absorbed from the small intestine rather than digested, and can lead to a luminal distension by various mechanisms (water fermentation, rapid fermentation, gas,…) [29,64,65]. In this study, vegetarians had higher intakes of both simple and complex carbohydrates.

Although lactose malabsorption does not appear to be a cause of IBS or to be more prevalent in individuals with IBS than in the general population [31,66–71], lactose is not well digested and absorbed by a majority of adults throughout the world, and individuals with and without IBS may report increased symptoms, similar to those of IBS, following ingestion of lactose-containing foods. Thus, the low lactose intakes of vegetarians could help to improve IBS symptoms. Overall, the vegetarian diet presents both features that might improve and worsen IBS symptoms. This may partly explain the absence of any significant association observed between VD and IBS.

We found a positive association between consistent VD and IBS (aOR = 2.58, 95%CI 1.36–4.87 with p = 0.004), IBS-M (aOR = 2.94, 95%CI 1.19–7.31 with p = 0.02) and IBS-D (aOR = 2.77, 95%CI 1.01–7.59 with p = 0.047). Similar results were shown by including vegans in the outcome, with in addition a significant association with IBS-D with VD (at least once). These results are in line with those of Ghoshal and colleagues [40], where a predominant vegetarian diet was associated with IBS (aOR = 10.77, 95%CI 1.49–77.89) in 2,774 subjects (including 2,654 vegetarians). However, these results should be interpreted carefully. Indeed, the low numbers of consistent vegetarians (n = 106) probably relates to a very particular population, whose dietary behavior and lifestyle can probably not be generalized to the entire vegetarian population [63].

Finally, and in accordance with previous studies, we observed that vegetarians had lower energy [72,73], sodium and SFA intakes, whereas they had higher intakes of PUFA and MUFA. These characteristics have been shown to reduce cardiovascular risk [13,60,74,75].

The identification of IBS was based on the Rome III criteria which was considered the gold standard at the time of inclusion [21]. The prevalence of IBS in our study is in agreement with other studies realized among the French population i.e.: 4% by Bommelaer and colleagues and 4.7% by Dapoigny and colleagues [76,77]. Our prevalence was slightly higher, which could partly be due to the modification of diagnosis criteria for IBS, former criteria tending to have higher detection rates compared to the Rome III [23]. Likewise, the proportion of vegetarians in our sample fits with the estimated proportion of vegetarians in France (about 2%) [1]. Finally, we used a Web-based dietary assessment which was compared with a traditional dietitian’s interview and showed a good agreement with this gold standard [78–80].

However some limitations should be discussed. This is a cross-sectional study, and although we excluded all subjects who declared they were vegetarian after filling-in the FGIDs questionnaire, we were not able to conclude on causality (i.e. determine if vegetarian diet tended to increase IBS symptoms or if participants with an IBS were more likely to adopt such a diet in order to improve their digestive symptoms). Another limitation is that subjects were recruited the general population and were volunteers. They were therefore more likely to be health conscious and have more controlled diets, and probably not representative of the general population. Nevertheless, the representativeness is not necessarily required in analytical studies [81], and the prevalence of IBS in our study was similar to that of the French population, which is not in favor of a selection bias. We observed that vegetarians had some meat and fish intakes in their dietary records. Indeed, we were not able to know precisely what kind of vegetarianism (e.g. ovolactovegetarianism vs. pescovegetarianism, semi-vegetarianism) was followed by self-declared vegetarian participants. Thus, it is possible that these people declared themselves vegetarian and then changed a part of their food habits to include some animal products. Moreover, a report performed by the Human Research Council on current and former vegetarians and vegans in the USA, have shown that almost 85% of vegetarians/vegans abandon their diet [82], mainly because maintaining this type of diet is difficult in the long run. Plus, a recent study performed among the general population in Belgium showed that semi-vegetarian (i.e. flexitarism) represented almost 12% of the surveyed population [9]. This is therefore consistent with our vegetarian population for whom self-reported vegetarianism can vary over time.

Further leads remain to be explored like the modifications of the gut microbiota related to the vegetarianism. Indeed, the composition of the gut microbiota has been shown to be responsive and adaptable to the diet of the host organism [83,84]. And recent works highlighted significant differences between the faecal microbiota of omnivores, vegetarians and vegan [85,86]. Finally, it could be appropriate to focus on (FODMAP’s) content of a usual vegetarian diet.

## Conclusion

Overall, vegetarian diet did not appear to be associated with IBS in our study, unless we find positive associations between a stable vegetarian diet and IBS (including IBS mixed and IBS diarrhoea). But more research is needed to assess the association between vegetarian diet and relief of symptoms of IBS patients so clinicians will be able to consider vegetarian diet as one of the treatment options for IBS.

## Supporting information

S1 Table Comparison between included and excluded subjects according to sociodemographic characteristics (N = 50,466). (DOCX)

S2 Table Comparison of food items (in g/day) according to the type of vegetarianism (n = 805). (DOCX)

S3 Table Comparison of macronutrients according to the type of vegetarianism (n = 805). (DOCX)

S4 Table Multivariate analysis (logistic regression models) (N = 41,682). (DOCX)

S5 Table Multivariate analysis including vegans (logistic regression models) (N = 41,908). (DOCX)

## Abbreviations

aOR: adjusted Odds Ratio
BMI: Body Mass Index
CU: Consumer Unit
FGID: Functional GastroIntestinal Disorder
FODMAPs: Fermentable Oligo-, Di-, Monosaccharides And Polyols
IBS: Irritable Bowel Syndrome
IBS-C: IBS Constipation
IBS-D: IBS diarrhoea
IBS-M: IBS Mixed
IBS-U: IBS Undefined
IPAQ: International Physical Activity Questionnaire
VD: Vegetarian Diet
95% CI: 95% Confidence Interval
