# Supplementary material for: Association between self-reported vegetarian diet and the irritable bowel syndrome in the French NutriNet cohort
Source: PLoS One. 2017 Aug 25;12(8):e0183039. doi: 10.1371/journal.pone.0183039 (PMC5571937; doi:10.1371/journal.pone.0183039)
Supplement: S1 Table — (DOCX) [file pone.0183039.s001.docx]

S1 table. Comparison between included and excluded subjects according to sociodemographic characteristics (N=50,466)

|  |  | Included N=41,682 | Removed N=8,784 | p value |
| --- | --- | --- | --- | --- |
| Gender (%) | Men | 22.0 | 27.3 | <0.0001 |
|  | Women | 78.0 | 72.7 |  |
| Age (mean ± SE) |  | 49.8 (+/-14.3) | 47.5 (+/-14.8) | <0.0001 |
| Educational level (%) | No diploma and primary studies | 2.9 | 2.7 | 0.07 |
|  | Secondary studies | 33.1 | 32.1 |  |
|  | High educational level | 63.9 | 65.2 |  |
| Income level (%) | <1200 € | 15.4 | 18.3 | <0.0001 |
|  | 1200-2300 € | 43.7 | 43.2 |  |
|  | > 2300 € | 41.0 | 38.6 |  |
| Smoking status (%) | Non smoker | 51.7 | 47.7 | <0.0001 |
|  | Former smoker | 35.3 | 36.6 |  |
|  | Current smoker | 13.0 | 15.7 |  |
| Marital status (%) | Single | 26.7 | 28.9 | <0.01 |
|  | Cohabiting | 73.3 | 71.1 |  |
| Physical activity | Low | 22.0 | 23.3 | 0.45 |
|  | Moderate | 42.9 | 42.5 |  |
|  | Intense | 35.0 | 34.3 |  |

Abbreviations: *SE: standard Error*

**Missing data**: Income level n=5349 (10.6%); Physical activity n= 6411 (12.7%)
