# Supplementary material for: Association between self-reported vegetarian diet and the irritable bowel syndrome in the French NutriNet cohort
Source: PLoS One. 2017 Aug 25;12(8):e0183039. doi: 10.1371/journal.pone.0183039 (PMC5571937; doi:10.1371/journal.pone.0183039)
Supplement: S2 Table — (DOCX) [file pone.0183039.s002.docx]

S2 table. Comparison of food items (in g/day) according to the type of vegetarianism (n=805)

|  | Vegetarians (n=699) | | Stable vegetarians (n=106) | |  |
| --- | --- | --- | --- | --- | --- |
|  | Mean | SE | Mean | SE | p |
| **Meat, poultry** | 37.1 | 2.5 | 14.2 | 4.7 | **<0.0001** |
| **Porc ham, poultry cuts, processed meat** | 17.0 | 1.2 | 6.5 | 2.2 | **<0.0001** |
| Fish, shellfish, processed fish and shellfish | 44.6 | 2.7 | 39.5 | 5.0 | 0.33 |
| Eggs | 21.1 | 1.2 | 18.4 | 2.2 | 0.23 |
| Milk, yogurt | 130.7 | 7.1 | 138.7 | 13.3 | 0.56 |
| Cheese, cottage cheese, Petits Suisses | 78.7 | 3.6 | 85.8 | 6.8 | 0.31 |
| Starchy food | 212.7 | 5.1 | 222.5 | 9.5 | 0.32 |
| Wholegrain products | 90.9 | 4.1 | 101.4 | 7.8 | 0.19 |
| Breakfast cereals | 44.9 | 2.1 | 49.9 | 4.0 | 0.22 |
| Dry fruits, oleaginous fruits | 17.3 | 1.3 | 19.9 | 2.4 | 0.30 |
| **Fruits** | 221.3 | 8.1 | 257.1 | 15.2 | **0.02** |
| **Vegetables** | 262.3 | 6.8 | 297.5 | 12.8 | **<0.01** |
| Pulses | 37.1 | 2.0 | 42.3 | 3.8 | 0.18 |
| 100% legumes and fruits juice | 76.1 | 4.4 | 85.1 | 8.3 | 0.29 |
| Condiments, spices | 11.8 | 0.6 | 10.7 | 1.1 | 0.31 |
| Oil | 11.0 | 0.4 | 11.1 | 0.8 | 0.89 |
| Non sugared beverages | 1193 | 30.9 | 1093 | 57.7 | 0.09 |
| Soft sugary drinks | 59.5 | 5.4 | 74.4 | 10.1 | 0.15 |
| Alcoholic beverages | 86.3 | 5.8 | 91.4 | 10.8 | 0.65 |
| Fat products | 35.4 | 1.1 | 36.2 | 2.0 | 0.69 |
| Fat and sugared products | 166.8 | 4.6 | 182 | 8.6 | 0.09 |
| Salty and sweet snack products | 21.3 | 1.1 | 21.3 | 2.1 | 1.00 |

Abbreviations: *SE: standard Error*
