# Supplementary material for: Association between self-reported vegetarian diet and the irritable bowel syndrome in the French NutriNet cohort
Source: PLoS One. 2017 Aug 25;12(8):e0183039. doi: 10.1371/journal.pone.0183039 (PMC5571937; doi:10.1371/journal.pone.0183039)
Supplement: S3 Table — (DOCX) [file pone.0183039.s003.docx]

S3 table. Comparison of macronutrients according to the type of vegetarianism (N=805)

|  | Vegetarians (n=699) | | Stable vegetarians (n=106) | |  |
| --- | --- | --- | --- | --- | --- |
|  | Mean | SE | Mean | SE | p |
| Energy (Kcal) | 1,977.8 | 20.4 | 1,964.9 | 39.6 | 0.75 |
| %energy from fat | 38.3 | 0.33 | 37.2 | 0.64 | 0.10 |
| MUFA (g) | 28.7 | 0.38 | 27.7 | 0.71 | 0.19 |
| Omega 3 | 1.4 | 0.04 | 1.3 | 0.07 | 0.19 |
| Omega 6 | 10.4 | 0.20 | 10.3 | 0.37 | 0.94 |
| PUFA (g) | 12.4 | 0.21 | 12.3 | 0.40 | 0.75 |
| SFA (g) | 29.0 | 0.40 | 28.2 | 0.74 | 0.29 |
| **Cholesterol (mg)** | 243.4 | 5.20 | 208.1 | 9.73 | **<0.001** |
| **%energy from protein** | 14.4 | 0.18 | 13.3 | 0.35 | **<0.01** |
| **Animal proteins (g)** | 35.0 | 0.88 | 28.6 | 1.64 | **<0.0001** |
| Vegetal proteins (g) | 30.6 | 0.44 | 32.3 | 0.82 | 0.05 |
| **%energy from carbohydrates** | 44.7 | 0.34 | 47.1 | 0.67 | **<0.001** |
| Complex carbohydrates (g) | 108.3 | 1.33 | 110.5 | 2.5 | 0.39 |
| **Simple carbohydrates (g)** | 93.4 | 1.30 | 100.9 | 2.4 | **<0.01** |
| **Fibers (g)** | 23.7 | 0.38 | 25.4 | 0.71 | **0.02** |

Abbreviations: *SE: standard Error*
