# Supplementary material for: Association between self-reported vegetarian diet and the irritable bowel syndrome in the French NutriNet cohort
Source: PLoS One. 2017 Aug 25;12(8):e0183039. doi: 10.1371/journal.pone.0183039 (PMC5571937; doi:10.1371/journal.pone.0183039)
Supplement: S4 Table — (DOCX) [file pone.0183039.s004.docx]

S4 table. Multivariate analysis (logistic regression models) (N=41,682)

|  | Omnivorous | Vegetarian (once or twice)  aOR [95%CI] | Vegetarians (three times or more)  aOR [95%CI] | p trend |
| --- | --- | --- | --- | --- |
| **IBS** | Ref. | 1.01 [0.71-1.44] | **2.61 [1.38-4.95]** | 0.06 |
| **IBS mixed** | Ref. | 1.04 [0.60-1.82] | 2.97 [1.20-7.37] | 0.11 |
| **IBS diarrhoea** | Ref. | 1.13 [0.63-2.01] | **2.78 [1.01-7.62]** | 0.12 |
| **IBS constipation** | Ref. | 0.86 [0.38-1.94] | 2.29 [0.56-9.41] | 0.69 |
| **IBS undefined** | Ref. | 0.86 [0.27-2.72] | NA | 0.54 |

*Models are adjusted for: Age, educational level, total energy intake, income level, smoking status, BMI, physical activity and gender*

*Abbreviations: IBS Irritable Bowel Syndrome; NA Not Applicable; OR Odds Ratio; 95%CI Confidence Interval*
