# Supplementary material for: Association between self-reported vegetarian diet and the irritable bowel syndrome in the French NutriNet cohort
Source: PLoS One. 2017 Aug 25;12(8):e0183039. doi: 10.1371/journal.pone.0183039 (PMC5571937; doi:10.1371/journal.pone.0183039)
Supplement: S5 Table — (DOCX) [file pone.0183039.s005.docx]

S5 table. Multivariate analysis including vegans (N=41,908).

|  | Omnivorous | Vegetarian (once or twice)  aOR [95%CI] | Vegetarians (three times or more)  aOR [95%CI] | p trend |
| --- | --- | --- | --- | --- |
| **IBS** | Ref. | 0.84 [0.57-1.22] | **2.23 [1.53-3.25]** | **0.01** |
| **IBS mixed** | Ref. | 1.10 [0.65-1.89] | 1.44 [0.71-2.93] | 0.13 |
| **IBS diarrhoea** | Ref. | 0.73 [0.36-1.48] | **3.54 [2.12-5.92]** | **<0.01** |
| **IBS constipation** | Ref. | 0.70 [0.29-1.69] | 2.04 [0.90-4.63] | 0.61 |
| **IBS undefined** | Ref. | 0.57 [0.14-2.31] | 0.63 [0.09-4.51] | 0.33 |

*Models are adjusted for: Age, educational level, total energy intake, income level, smoking status, BMI, physical activity and gender*

*Abbreviations: IBS Irritable Bowel Syndrome; NA Not Applicable; OR Odds Ratio; 95%CI Confidence Interval*
